# Supplementary material for: Score-Based Image-to-Image Brownian Bridge
Source: Proc ACM Int Conf Multimed. Author manuscript; Available in PMC 2025 Apr 8. (PMC11977112; doi:10.1145/3664647.3680999)
Supplement: supplemental [file NIHMS2070238-supplement-supplemental.pdf]

Supplementary Materials: The Name of the Title is Hope

Anonymous Authors

1 PROOF OF THEOREM 1

Suppose the SDE in Eq. 1 is amenable with $\mathbf{u}(X)$ and $M(t)$ satisfying (2) and (3). Applying the Itô's formula, we have for each k

$$d\left(\frac{u^k(X_t)}{1-t}\right) = \left(\frac{u^k}{(1-t)^2} - \frac{\nabla u^k \cdot \mathbf{F}(X_t)}{(1-t)^2} + \frac{1}{2(1-t)}\operatorname{tr}\left(G(X_t,t)^T D^2 u^k G(X_t,t)\right)\right) dt + \frac{\nabla u^k \cdot G(X_t,t)}{1-t}\, dW$$

The amenability condition Eq. 3 implies that

$$d\left(\frac{u^k(X_t)}{1-t}\right) = \frac{M^k(t)}{1-t}\, dW.$$

The functional equation Eq. 4 follows from integrating the above equation over $[0,t]$.

From Eq. 4, we find the covariance matrix of $\mathbf{u}(X_t)$ is

$$(1-t)^2 \int_0^t \frac{M(\tau)M(\tau)^T}{(1-\tau)^2}\, d\tau$$

which converges to 0 as $t \to 0-$. This fact and the amenability condition in Eq. 2 jointly lead to the conclusion that

$$\lim_{t\to 1-} X_t = \mathbf{0} \ \text{ almost surely.}$$

2 JUSTIFICATION OF AMENABILITY OF THE LINEAR A-BRIDGE

In the linear A-Bridge (Eq. 6), $\mathbf{F} = X - Y$ and $\mathbf{G} = \lambda\sqrt{1-t}I$. Take $\mathbf{u}(X) = X - Y$. Then $D\mathbf{u}(X) = I$, and $D^2\mathbf{u} = \mathbf{0}$. The both sides of the first equation in Eq. 3 is equal to $X - Y$, while the left-hand-side of the second is equal to $\lambda\sqrt{1-t}I$ which depends on t only.

3 ADDITIONAL QUALITATIVE COMPARISON

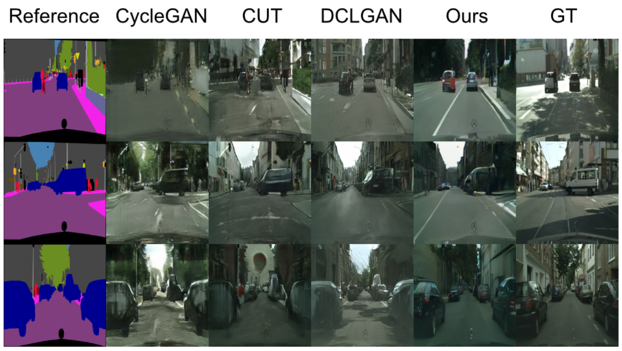

Figure 1: Additional qualitative comparison between our linear A-Bridge and other methods on the Cityscapes datasets. GT stands for Ground Truth.

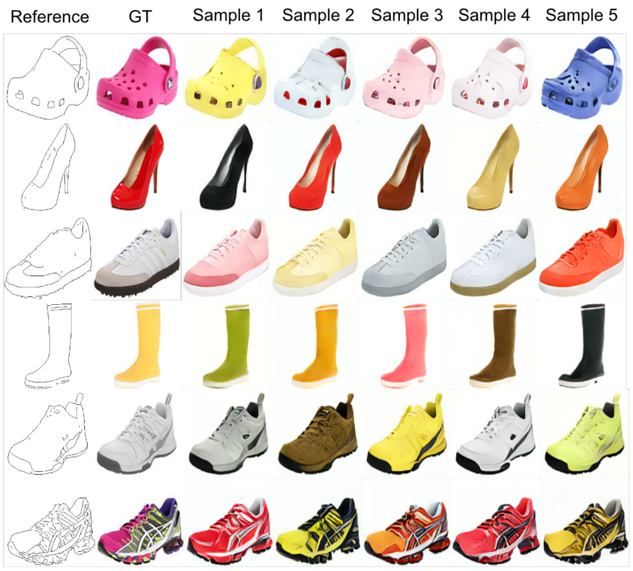

Figure 2: Additional diversity samples of our linear A-Bridge on the Edges2Shoes dataset.

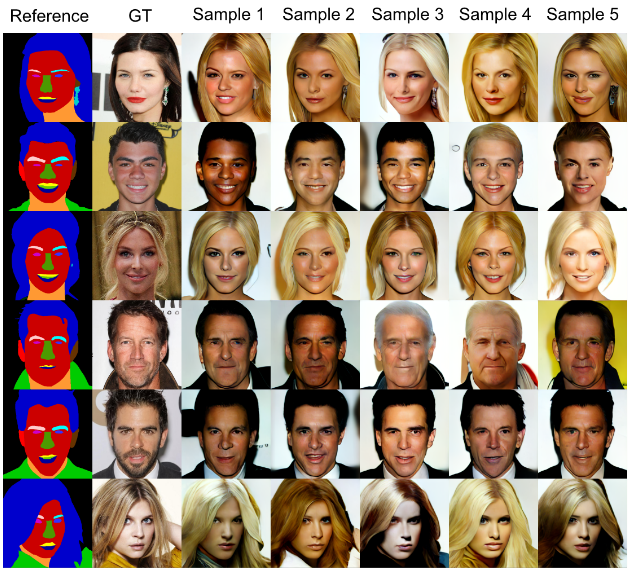

Figure 3: Additional diversity samples of our linear A-Bridge on the CelebAMask-HQ dataset.

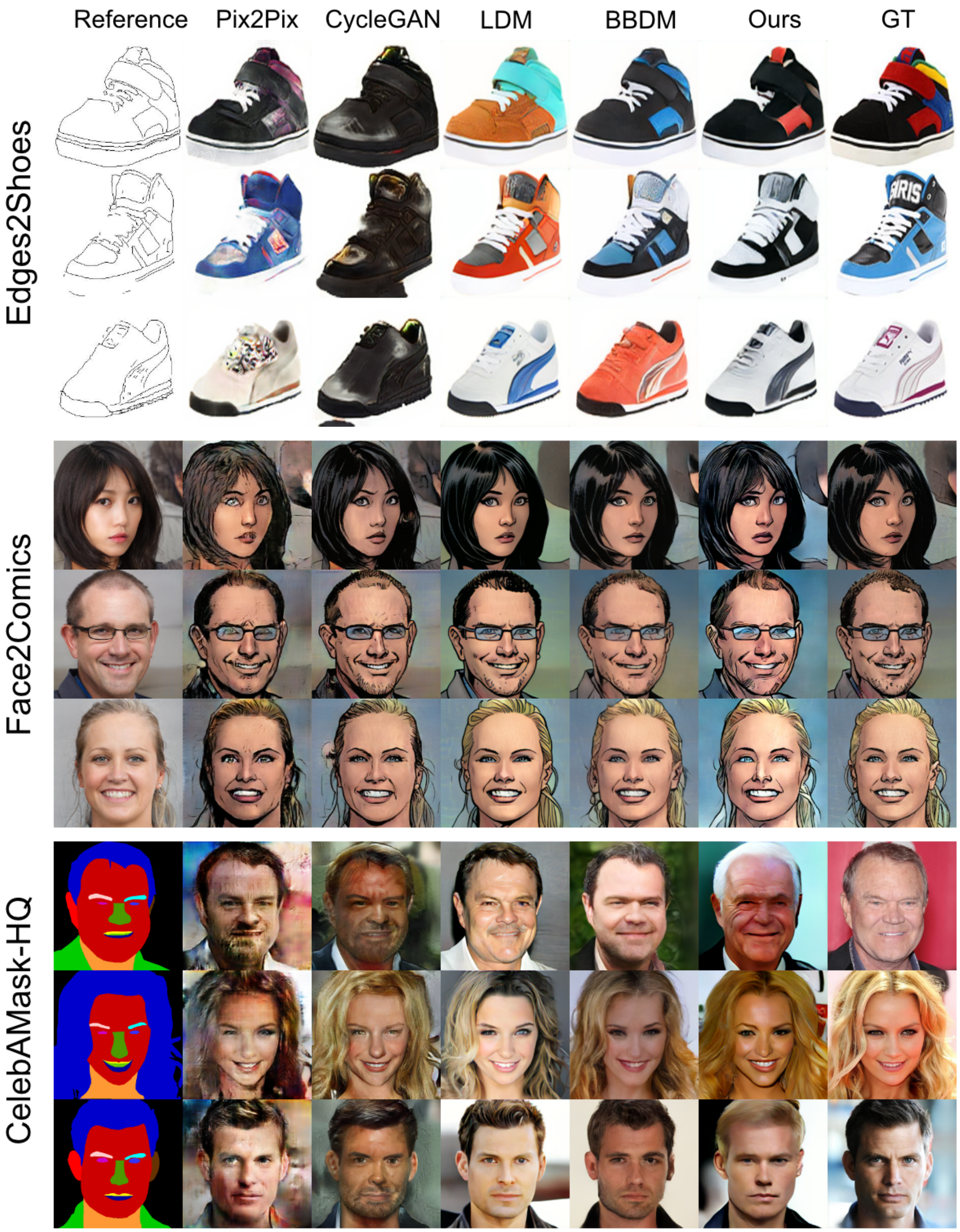

Figure 4: Additional qualitative comparison between our linear A-Bridge and other methods on the Edges2Shoes, Face2Comics, and CelebAMask-HQ datasets. GT stands for Ground Truth.
